# Does Health Literacy Make a Difference? Comparing the Effect of Conventional Medicine Versus Homeopathic Prescribing on Treatment Credibility and Expectancy

**DOI:** 10.3389/fpsyg.2021.581255

**Published:** 2021-06-01

**Authors:** Marcel Wilhelm, Frank Euteneuer

**Affiliations:** ^1^Department of Clinical Psychology and Psychotherapy, Philipps University of Marburg, Marburg, Germany; ^2^Department of Psychology, Clinical Psychology and Psychotherapy, Medical School Berlin, Berlin, Germany

**Keywords:** homeopathic remedies, treatment credibility, treatment expectancy, health literacy, expectations

## Abstract

**Objective:**

While homeopathic remedies are often used to treat non-specific complaints such as headaches, empirical evidence suggests their treatment effect is due to the placebo effect. Low health literacy seems to be connected to higher use of complementary and alternative medicine (CAM). The aim of this study was to examine what people with occasional headaches expect from conventional medicine or homeopathic remedies and if health literacy interacts with this expectation.

**Methods:**

In this experimental study, *n* = 582 participants with occasional headaches were randomized to read one of two vignettes, which described the prescription of either conventional medicine or a homeopathic remedy. Subsequently, the participants were asked to rate treatment credibility and expectancy with regard to their assigned vignette. Health literacy was assessed as a potential moderator.

**Results:**

Participants in the conventional medicine group rated treatment credibility and expectancy higher than in the homeopathic remedy group. Moderation analysis revealed that when being offered conventional medicine, participant reports of treatment credibility and expectancy decreased with lower health literacy, while these outcomes increased with lower health literacy for homeopathic remedies.

**Discussion:**

People with occasional headaches estimate the effectiveness of conventional medication properly. However, health care professionals should pay special attention to patients with low health literacy, as they might need more time and information to give their informed consent.

## Introduction

Homeopathy, as a component of complementary and alternative medicine (CAM), is widely used by the general population (Relton et al., 2017). Homeopathy use worldwide ranges from 0.7 to 9.8% (12-month prevalence; median: 3.9%). Empirical evidence suggests that homeopathy does not work beyond the placebo effect (Antonelli and Donelli, 2018). This leads to an ethical dilemma: the prescription of homeopathic remedies can be seen as deceptive, when it is recommended as an effective medication (Shaw, 2015). However, prescribers of homeopathy do not actively deceive patients as long as they believe in its effect themselves (Levy et al., 2015). The data show that general practitioners are usually aware of the placebo effect and use a variety of “impure” placebos (substances with potential pharmacological effect; e.g., off-label medication, CAM such as homeopathy, vitamin preparations) in order to achieve psychological treatment effects or for non-specific complaints (Meissner et al., 2012; Howick et al., 2013).

Homeopathy can be dangerous, especially when it replaces evidence-based drugs in severe conditions, such as curable cancer (Johnson et al., 2018b). Even complementary use of homeopathy is associated with a higher risk of death and a higher refusal rate of conventional cancer therapy (Johnson et al., 2018a). The literature on the financial implications of adding homeopathic remedies is ambiguous. Whether costs are reduced or increased seems to depend on the health condition and the studies undertaken (Colas et al., 2015; Ostermann et al., 2015; Kass et al., 2020).

Why do patients use homeopathic remedies? As a placebo treatment, the most important predictor for the effect of homeopathy is treatment expectations (Sanders et al., 2020), which are also an essential part of analgesic drug effects (Bingel et al., 2011). A warm, trustworthy and empathic doctor-patient-communication is another very important mechanism to foster placebo effects (Schedlowski et al., 2015; Howe et al., 2017). It is also more crucial for clinical benefits of homeopathy than the remedy itself (Brien et al., 2011). Analgesic placebo effects regarding different types of headaches are well proven (de Craen et al., 2000; Nilsson Remahl et al., 2003; Steiner et al., 2003; Macedo et al., 2006; Locher et al., 2017). Headaches are highly prevalent (Stovner and Andree, 2010) and frequently treated with homeopathy (Dossett and Yeh, 2018). Additionally, low health literacy, i.e., the ability to communicate, process, and comprehend basic health information, can lead to unrealistic (Smith et al., 2009) or negative treatment expectations (Hadden et al., 2018) and is hypothesized to be linked to high CAM use (von Conrady and Bonney, 2017).

The aim of this study was thus to examine what people with occasional headaches expect from conventional medicine or homeopathic remedies and in what way health literacy interacts with this expectation.

## Materials and Methods

### Participants

Participants (*n* = 582) were recruited via online social network groups and established university mailing lists. Informed consent was obtained from all participants before participation. The study design complied with the principles of Helsinki and was approved by the Institutional Review Board of the Department of Psychology, University of Marburg. Assessment took place via an online survey using https://www.soscisurvey.de. Participants (aged ≥ 18 years) were eligible to take part in the study if they experienced (at least) occasional headaches. Before starting the experiment, participants completed questionnaires assessing baseline characteristics, information about previous use of analgesics and homeopathic remedies, as well as health literacy.

### Experimental Design

Participants were randomly assigned via Sosci Survey’s random generator function to read one of two vignettes. Randomization was done directly after agreeing to and signing the study’s informed consent form. The potential scenario in both vignettes was a medical doctor-patient consultation; no traditional homeopath-patient consultation scenario was used. Both vignettes started with the following instruction: “*Imagine that lately, you have suffered from occasional headaches. These occur from time to time during the day. The pain is sometimes very uncomfortable and affects your everyday life activities. No matter what you do, the headache keeps coming back. You begin to wish that the headache would subside so that you can manage your life without being affected by pain. You therefore decide to see a doctor and ask for help. You describe your symptoms to the doctor and state that you would like a drug for your headaches. After a detailed discussion and a physical exam, the doctor explains that your headaches are not due to a serious physical illness.*” The vignette for the conventional medicine condition continued as follows: “*He explains to you: I prescribe Ibuprofen, which you take when you experience a headache. It is a widely used and well-known drug for various types of headaches. It is taken in the form of pills. This treatment has helped many of my patients, they feel better now. If your headache occurs, you should take 1–2 tablets.*” In contrast, the vignette for the homeopathic remedy condition continued as follows: “*He explains to you: I prescribe Belladonna, which you take when you experience a headache. It is a widely used and well-known homeopathic remedy for various types of headache. It is taken in the form of globules. This treatment has helped many of my patients, they feel better now. If your headache occurs, you should take five globules.*” Subsequently, participants were asked to rate treatment credibility and expectancy.

### Measurement Instruments

The Pain Disability Index (PDI; Tait et al., 1987) was used to measure pain-related disability. The seven items of the PDI range from 0 (no disability) to 10 (total disability). The sum of the seven items ranges from 0 to 70, with higher scores reflecting higher interference of pain with daily activities.

Health literacy was assessed with the short form of the European Health Literacy Questionnaire (HLS EU-Q16; Röthlin et al., 2013). This questionnaire consists of 16 items ranging from 1 (fairly easy) to 4 (very difficult), which assess the ability to access, understand, appraise and apply health information. An overall HLS EU-Q16 index was calculated following the manual for the instrument (Röthlin et al., 2013). For this purpose, item scores were dichotomized, and a sum score was calculated ranging from 0 to 16. Higher scores indicate higher health literacy.

Treatment credibility and expectancy were assessed using the Credibility/Expectancy Questionnaire (CEQ; Devilly and Borkovec, 2000). The subscale for credibility captures how believable, convincing, and logical the treatment is, and the subscale for expectancy assesses how much the participants expect that the treatment will alleviate their headache. Each subscale consists of three items ranging from 1 or 0% (logical/useful/confident) to 9 or 100% (very logical/useful/confident), depending upon the item. The total score ranges from 2 to 27 where higher scores indicate higher credibility and expectancy, respectively.

### Statistical Analysis

Statistical analysis was carried out with Mplus7 (Muthén and Muthén, 2012) and IBM SPSS version 23.0 for Windows (Chicago, SPSS, Inc.). Group differences were assessed using *t*-tests (if necessary, with Welch’s correction). A path analysis with full information maximum likelihood estimation and 10,000 bootstraps was performed to simultaneously examine lower order effects and the interaction effect between settings (i.e., conventional medicine vs. homeopathy) and health literacy on both outcomes (i.e., treatment credibility, treatment expectancy).

## Results

Mean age of the 582 participants was 35.96 years (*SD* = 13.86, range: 18 –95 years). The sample consisted of 472 women (81.10%) and most participants had upper secondary school education (74.78% with upper secondary school education). There were no significant differences in participant characteristics between the two experimental groups prior to the reading of vignettes. Table 1 shows sample characteristics for baseline variables and outcome measures separated by group.

**TABLE 1:** Sample characteristics.

**Variables**	**Conventional medicine (*n* = 292)**	**Homeopathy (*n* = 290)**	***p***
Age, years	35.47 (13.57)	36.46 (14.14)	0.386
Females, *n* (%)	239 (81.8)	233 (80.3)	0.301
Lower/Upper Secondary school education^1^	62/223	53/233	0.349
Pain Disability Index	25.01 (17.02)	24.49 (16.67)	0.706
Use of pain agents if required			
Conventional analgesics, *n* (*%*)	243 (83.2)	235 (81.0)	0.517
Homeopathic remedies, *n* (*%*)	18 (0.1)	25 (0.1)	0.271
Health Literacy	11.01 (3.28)	11.29 (3.29)	0.318
Post-manipulation treatment credibility	14.56 (6.28)	11.13 (7.08)	<0.001
Post-manipulation treatment expectancy	14.03 (6.90)	10.50 (7.17)	<0.001

*Values shown as mean (SD) unless otherwise noted. Group differences were calculated using *t*-tests and χ2 tests as appropriate.^1^Lower secondary education refers to German school leaving examinations from a general secondary school [*Hauptschulabschluss*] or a secondary school [*Mittlere Reife*]; upper secondary education refers to a range of German school leaving examinations which enable university entrance [*Fachhochschulreife, fachgebundene Hochschulreife, allgemeine Hochschulreife*].*

**FIGURE 1:**
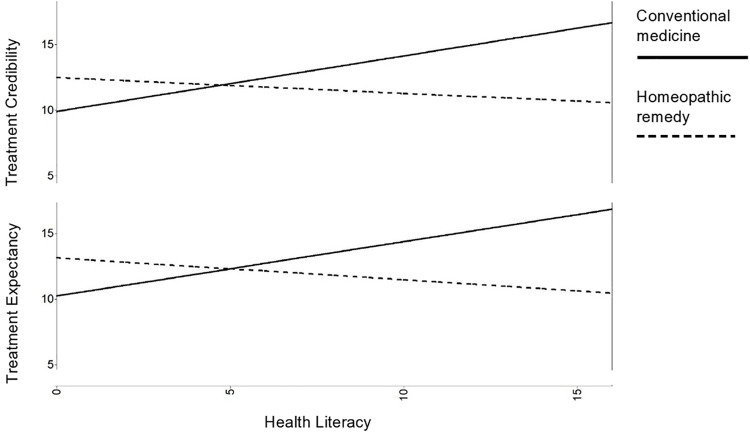
Interaction between health literacy and treatment condition. The superiority of conventional medicine offers over homeopathic remedy offers decreases with lower health literacy.

Compared to individuals asked to imagine being offered conventional medicine, participants asked to imagine being offered a homeopathic remedy reported lower levels of treatment credibility, *t*(570.96) = 6.16, *p* < 0.001, and treatment expectancy, *t*(580) = 6.06, *p* < 0.001. However, health literacy had an impact on group differences in treatment credibility (β = –0.61, *p* = 0.001) and treatment expectancy (β = –0.62, *p* = 0.001). Reported treatment credibility and expectancy for participants asked to imagine being offered conventional medicine decreased with lower health literacy, while they increased with lower health literacy for participants asked to imagine being offered homeopathic remedies. Thus, the perceived superiority of conventional medicine offers over homeopathic remedy offers with respect to treatment credibility and expectancy increased with higher health literacy. Figure 1 plots these interactions.

## Discussion

In this study, participants in the conventional medicine group rated treatment credibility and expectancy higher compared to participants in the homeopathic remedy group. In the conventional medicine group, participant reports of treatment credibility and expectancy decreased with lower health literacy, while these outcomes increased with lower health literacy in the homeopathic remedy group. Thus, the perceived superiority of conventional medicine offers over homeopathic remedy offers regarding treatment credibility and expectancy increased with higher health literacy.

The results appear to be robust, as the large sample matches the prevalence of headaches with predominantly female participants and a mean age between 20 and 50 years and a substantial pain disability due to occasional headaches (Stovner and Andree, 2010). Participants only received one vignette and were not aware of another treatment or group. Therefore, the data are not confounded by intraindividual comparisons between the two treatment vignettes.

These results show that people with high health literacy tend to expect less relief from homeopathic remedies than those with low health literacy, and they emphasize the importance of identifying patients with low health literacy early in the treatment process. These patients tend to need more attention and explanation but tend to receive less time during doctor visits (Menendez et al., 2016). Improved communication by physicians is therefore necessary to inform these patients more comprehensively. Homeopaths match these needs, as their communication style is more participatory and allows longer consultations (Marian et al., 2008). Doctor–patient-communication is of importance for subjective and objective health outcomes (Riedl and Schüßler, 2017). Conventional medical treatments must improve – not in terms of consultation length *per se*, but in terms of quality of the participatory engagement as a key part of a successful doctor-patient-relationship (Ha, 2010).

A remaining issue is if patients with low health literacy can give informed consent, without being explicitly informed about the placebo-like character of the treatment. There is broad evidence that open-label placebos work (Kaptchuk and Miller, 2018). Giving a plausible (placebo) treatment rationale is more important than deception (Locher et al., 2017). Describing homeopathic remedies as placebo treatments should therefore be possible without the fear of losing its effect. Additionally, identifying low health literacy patients and supporting them, e.g., via health education programs, health communication trainings, or extended doctor visits, should be a focus of future research.

A few limitations should be discussed. The setting of both vignettes resemble situations of conventional medicine prescriptions and might therefore be in favor of the conventional medicine group. Consultation time of a homeopathic physician can take twice as long as the consultation time of a conventional physician (Bishop et al., 2007; Marian et al., 2008). The situations were only described via text. However, when used appropriately, vignette studies show satisfying internal, external, and construct validity (Evans et al., 2015). On the other hand, two more vignettes using a homeopathic setting could have provided more information. But as the doctor-patient-communication is a very important ingredient of homeopathic treatment (Brien et al., 2011), the text based vignettes could still have disadvantaged homeopathic prescribing. Future studies should use real interactions or video sessions to address this issue. While this limitation might have influenced the intergroup effect in favor of the conventional medicine group, the interaction with health literacy appears even more striking. The results should still be interpreted with caution, as a high level of education and the high number of female participants could have affected them.

In conclusion, individuals seem to be able to properly estimate the effectiveness of evidence-based conventional medication, unless their health literacy is low. Spending more consultation time with patients to explain treatment options without any deception is an important responsibility for health care professionals.

## Data Availability Statement

The datasets presented in this article are not readily available due to missing consent of the participants. Requests to access the datasets should be directed to Dr. Marcel Wilhelm (Email: marcel.wilhelm@uni-marburg.de).

## Ethics Statement

The studies involving human participants were reviewed and approved by Ethics Committee of the Department of Psychology, University of Marburg. The patients/participants provided their written informed consent to participate in this study.

## Author Contributions

Both authors listed have made a substantial, direct and intellectual contribution to the work, and approved it for publication.

## Conflict of Interest

The authors declare that the research was conducted in the absence of any commercial or financial relationships that could be construed as a potential conflict of interest.
